# Chondrosarcoma: With Updates on Molecular Genetics

**DOI:** 10.1155/2011/405437

**Published:** 2011-02-15

**Authors:** Mi-Jung Kim, Kyung-Ja Cho, Alberto G. Ayala, Jae Y. Ro

**Affiliations:** ^1^Department of Pathology, Asan Medical Center, University of Ulsan College of Medicine, Seoul 138-736, Republic of Korea; ^2^Department of Pathology, Weill Medical College of Cornell University, The Methodist Hospital, Houston, TX 77030, USA

## Abstract

Chondrosarcoma (CHS) is a malignant cartilage-forming tumor and usually occurs within the medullary canal of long bones and pelvic bones. Based on the morphologic feature alone, a correct diangosis of CHS may be difficult, Therefore, correlation of radiological and clinicopathological features is mandatory in the diagnosis of CHS. The prognosis of CHS is closely related to histologic grading, however, histologic grading may be subjective with high inter-observer variability. In this paper, we present histologic grading system and clinicopathological and radiological findings of conventional CHS. Subtypes of CHSs, such as dedifferentiated, mesenchymal, and clear cell CHSs are also presented. In addition, we introduce updated cytogenetic and molecular genetic findings to expand our understanding of CHS biology. New markers of cell differentiation, proliferation, and cell signaling might offer important therapeutic and prognostic information in near future.

## 1. Introduction

Chondrosarcoma (CHS) is a rare malignant tumor that produces cartilage matrix. The estimated overall incidence of CHSs is 1 in 200,000 per year [1], and it is the third most frequent malignant bone tumor after multiple myeloma and osteosarcoma. It is estimated that CHSs account for approximately 3.6% of the annual incidence of all primary bone malignancies in the USA [2] and 20~30% of primary malignant bone tumors [3].

CHSs that arise de novo are called primary CHSs, whereas CHSs developing superimposed on preexisting benign cartilage tumors such as an enchondroma or osteochondroma are referred to as secondary CHSs. CHSs are a heterogeneous group of tumors that can be categorized by anatomic location as central when they occur within the medullary canal or peripheral when they occur in the cartilage cap of an exostosis. In addition to conventional CHSs that show hyaline cartilage differentiation, there are other types of CHSs such as dedifferentiated, mesenchymal, or clear cell, subtypes which show distinct genetic and clinicopathologic characteristics [4] (Tables 1 and 2). Myxoid CHS is not included in this paper because its existence in bone is highly controversial. 

Most (about 85%) of CHSs, however, are of conventional CHSs, and the majority arises in the medullary cavity of long bone. The minority (up to 15%) of conventional CHSs is secondary peripheral CHSs which develop from the surface of bone as a result of malignant transformation within the cartilage cap of a preexisting benign cartilage tumor such as osteochondroma or develop de novo on the bone surface, and these are not uncommonly referred as juxtacortical or periosteal CHSs.

## 2. Conventional Intramedullary CHSs

### 2.1. Epidemiology

 Conventional CHS of bone is the most common type of primary CHS. Primary CHS typically affects an old population. The majority of patients are older than 50 years. The peak incidence is in the fifth to seventh decades of life. There is a male predilection of 1.5–2 to 1 [1].

### 2.2. Sites of Involvement

CHS can involve any bone; the incidence of axial and appendicular involvement is very similar. The bones of the pelvis, especially for ilium, are frequently involved. Long tubular bones are frequently affected. The proximal femur is the most preferred site, followed in frequency by proximal humerus, distal femur, and ribs. Other less frequently involved bones are the spine, scapula, and sternum. CHS rarely involves craniofacial bones, neck, forearm, clavicle, and sesamoids (including the patella). CHS in the small tubular bones is extremely rare (1~4% of all cases) [3, 5, 6].

### 2.3. Clinical Features and Imaging

Clinical symptoms are mostly nonspecific. Localized pain is the most frequent presenting symptom (about 80%) after local swelling. The symptoms are usually insidious, progressive, and worse at night and have a long duration (several months or years). Pathologic fractures are also common at initial presentation (up to 27%). 

**Figure 1:**
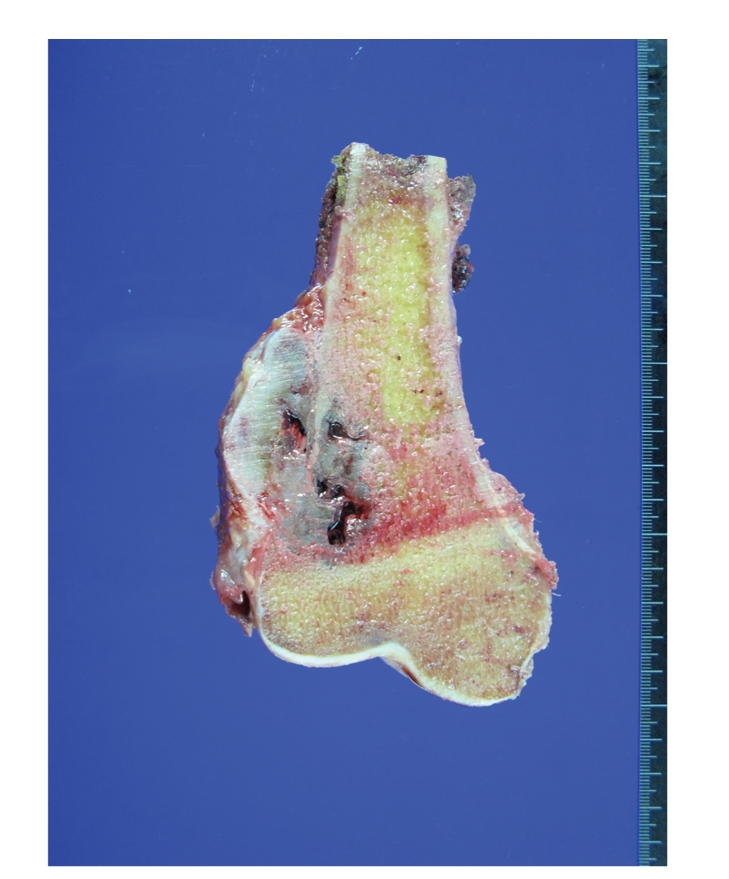
A well-demarcated cartilaginous tumor, measuring 6.5 cm in greatest dimension, in the metaphysis of distal femur. The mass extends through the periosteum to adjacent soft tissue.

Radiographs of conventional CHS typically reveal a mixed lytic and sclerotic pattern with characteristic small calcifications, often referred as “popcorn” or “ringlets” calcifications. In the long bones, primary CHS most commonly involves the metaphysis (49%), followed by the diaphysis (36%) (Figure 1). The presence of typical calcifications is radiologically diagnostic of cartilage, but often does not discriminate between benign, borderline, or malignant types of lesions. Size of the lesions (<5 cm), lack of break through the cortex, lack of infiltrative pattern, and lack of lytic component favors a benign or borderline process, while location in the axial skeleton and size greater than 5 cm are reliable predictor of low-grade CHS [7]. Radiographic findings including cortical destruction, soft tissue extension, and permeative changes such as the “moth-eaten pattern” are commonly associated to malignancy. A permeative pattern is often seen with high-grade CHS. Endosteal scalloping is a sign of aggressiveness in intramedullary cartilaginous lesions, but it is not completely diagnostic of malignancy. According to Murphey et al. [3], endosteal scalloping greater than two-thirds of the normal thickness of the long bone cortex is strong evidence of CHS over enchondroma. Thus, enchondromas and intramedullary low-grade CHSs (borderline tumors) of long bones often share similar radiological features. These lesions should be diagnosed by histologic examination after a complete resection of the lesion is made whether it is an excision or a complete curettage. Magnetic resonance (MR) imaging is also a preferred modality in diagnosis of cartilaginous tumor as well as in evaluation of the extent of marrow involvement and presence of soft tissue extension. On T1-weighted images after gadolinium contrast injection, marked “septal” or “ring-and-arc” enhancement is typical for enchondromas and low-grade CHSs which corresponds to fibrous bands between the confluent cartilage lobules on the histologic analysis. Inhomogeneous or homogeneous enhancement of high-grade CHSs correlates with high cellular areas on the microscopic examination [8]. In addition, fast contrast-enhanced MR imaging could assist in differentiation between enchondromas and CHSs. In the adult patient, both early and exponential enhancement is predictors of CHSs [9].

### 2.4. Pathology

**Figure 2:**
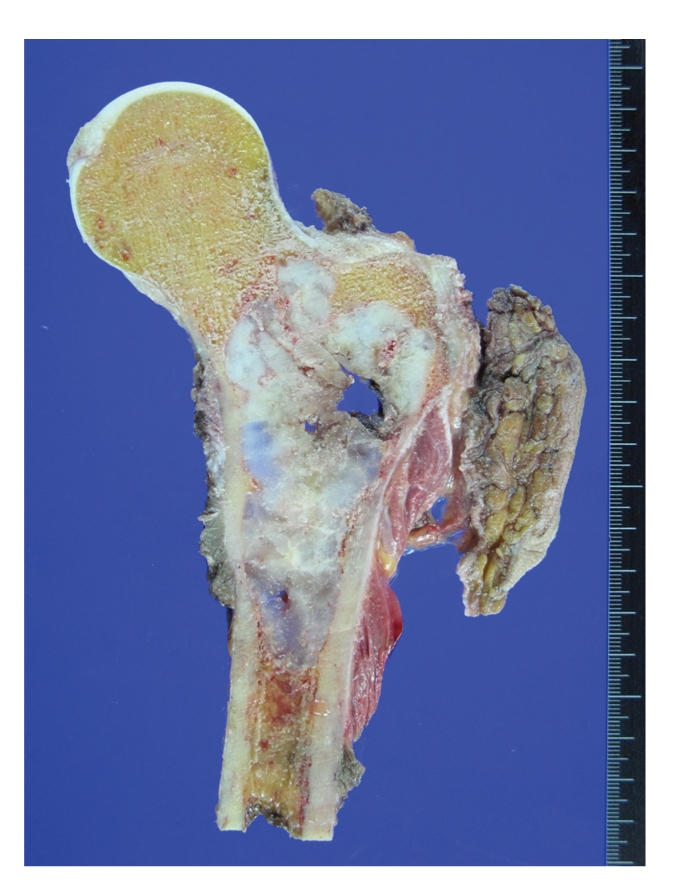
An intramedullary cartilaginous mass, measuring 9.5 cm in greatest dimension, in the metadiaphysis of proximal femur. Note the white to bluish gray and lobulated cut surface and cortical destruction.

**Figure 3:**
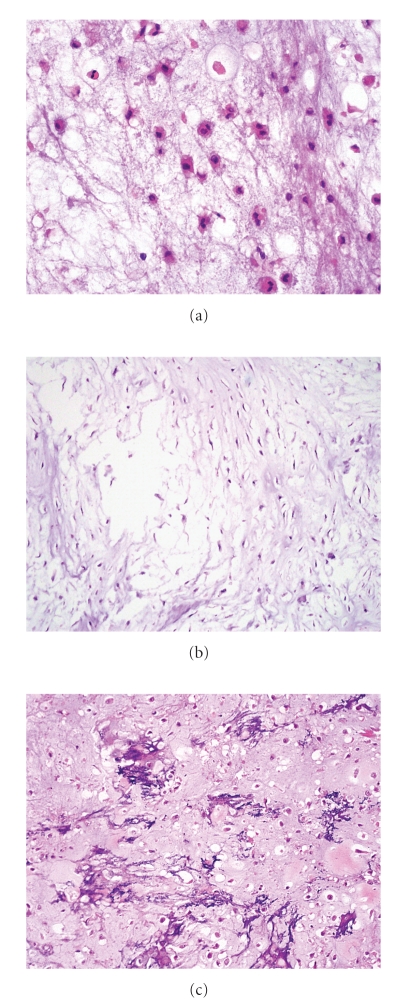
(a) Atypical chondrocytes with binucleation and eosinophilic cytoplasm; (b) chondrosarcoma with myxoid change; (c) chondrosarcoma with necrotic tumor cells and calcification.

 Conventional intramedullary CHSs are large lesions, usually greater than 4 cm in size [3]. CHSs grow in a lobulated pattern and are usually firm but may be soft, mucoid, or even gelatinous (Figure 2). They are white or bluish gray and often focally gritty because of matrix calcifications. The presence of gray hemorrhagic fish fleshy tissue or myxoid change is not uncommonly associated to a high-grade lesion. Histologic examination reveals lobules of hyaline cartilage with variable degrees of cellularity, myxoid change, and calcification. The chondrocytes usually have enlarged, hyperchromatic nuclei with binucleation (Figures 3(a)–3(c)). Necrosis and mitoses are mostly seen in high-grade lesions. 

#### 2.4.1. Grading

The grading is primarily based on nuclear size, hyperchromasia, cellularity, and mitoses [10]. The nuclear size is evaluated by assessing the tumor cells whether those cells are small and dark staining, moderate sized with visible intranuclear detail, or large and pleomorphic. The background is considered chondroid if definite lacunae are observed and myxoid if the cells are separated by basophilic intercellular substance without definite lacunae. 

**Figure 4:**
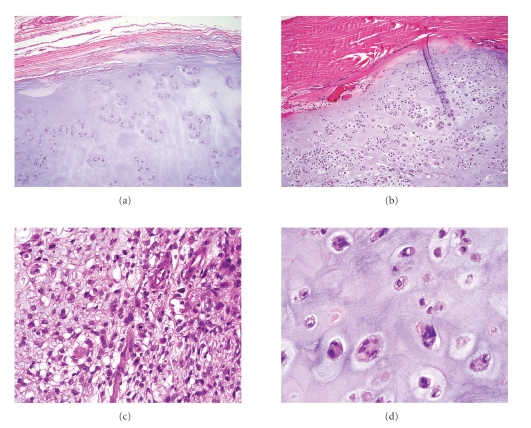
(a) Grade 1 CHS with chondroid matrix and low cellularity. Note the soft tissue extension. (b) Grade 2 CHS with increased cellularity and soft tissue extension. (c) Grade 3 CHS with more increased cellularity and cellular atypia. (d) Pleomorphic tumor cells and frequent multinucleated cells in grade 3 CHS.

**Figure 5:**
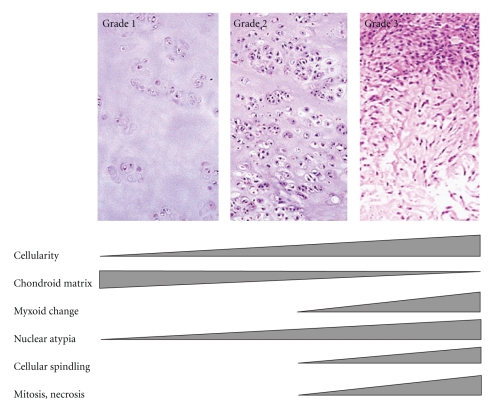
Schematic representation illustrating grading system of conventional CHS (not all CHSs follow this scheme).

Grade 1 (low-grade) lesions are poorly cellular with hyperchromatic round nuclei the size of a mature lymphocyte. There are no mitotic figures, or nuclear atypia and the cells retain the lacunar pattern. Myxoid background is not present, but there may be some degenerative myxoid change. Binucleated cells are rare if any. Dense cellularity, presence of significant numbers of moderately sized or larger nuclei, and mitotic figures are not features of low-grade CHS and, if present, indicate a higher grade of CHS [10]. Grade 2 (intermediate) tumors are more cellular lesions characterized by cells with nuclear enlargement; the chromatin may be fine with presence of nucleoli, and mitotic activity is rarely present. The cells also retain the lacunar pattern, and there is no myxoid change, although degenerative myxoid may be present. When myxoid stroma appears, this is clue that the tumor may become aggressive or is frankly malignant especially if it is associated to mitotic activity. Grade 3 (high-grade) tumors characteristically display 2 or more mitoses per ten high-power fields in the most cellular areas. There is usually a myxoid background associated to spindle or pleomorphic cells and the lacunar pattern is predominantly lost. Foci of necrosis are usually seen. For the purpose of clarification, myxoid change may associate to malignancy in cartilage tumors or may be degenerative; the latter is characterized by the presence of myxoid areas without cellularity, while the myxoid change associated to malignancy is characterized by a tumor without lacunar pattern with atypical spindle or stellate cells floating in a myxoid stroma (Figures 4(a)–4(d) and 5) [1, 3].

#### 2.4.2. Differential Diagnosis

**Figure 6:**
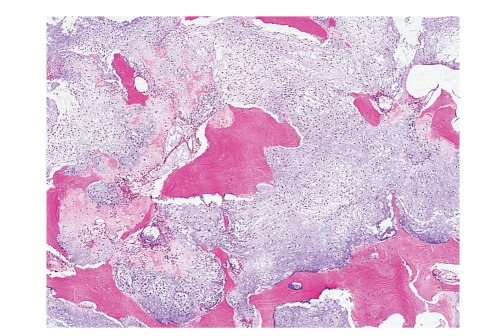
CHS permeating between preexisting bony trabeculae. Based on the cellularity and nuclear atypia, this lesion corresponds to grade 2 CHS.

**Figure 7:**
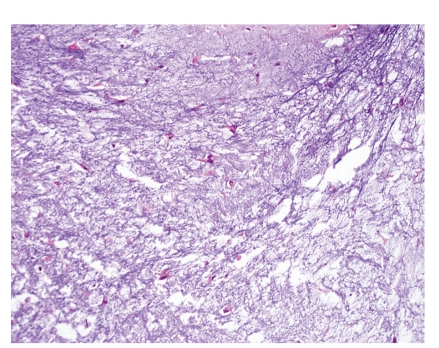
Extensive myxoid change in CHS.

More than 90% of conventional CHSs are low- to intermediate-grade tumors and should be distinguished from enchondroma. Permeation of cortical bone and/or preexisting medullary bone is most important to distinguish CHSs from enchondromas [11], for which it is crucial to take biopsy material consisting of cortical and medullary bones, and one should observe the growth pattern (Figure 6). One should avoid misinterpreting enchondroma as CHS by regarding areas of cartilage crushed into surrounding marrow spaces as true permeation in curettage specimen. The frequency of cellularity, double nuclei, and mitoses is similar between enchondroma and low-grade CHS. Recent study suggests that presence of myxoid matrix ≥20% and/or host bone entrapment strongly suggests CHS [12]. Extensive myxoid change is an ominous sign in a chondroid lesion, and in such cases one should try to search for other histologic features suggesting CHS (Figure 7). If one sees areas with undoubtful neoplastic osteoid, the lesion should be considered osteosarcoma with chondroblastic differentiation.

### 2.5. Genetics

So far, several attempts have been made to identify reliable molecular markers and therapeutic targets for CHS [13]. Collagen subtype has been proposed to reflect differentiation of CHS, specifically collagen types II and X as well as the proteoglycan aggrecan as a marker for mature neoplastic phenotype and collagen type I as a marker representing proliferative, “dedifferentiated” phenotype [14]. Cyclooxygenase-2 overexpression was also proposed as a marker associated with histologic grade and poor survival [15]. However, none of these biologic markers has been proved to provide independent prognostic information. The attempt to assess the effect of celecoxib (Celebrex) on CHS growth using xenograft model did fail to attain a satisfactory result [16]. Hedgehog signaling pathway that is important for development of central CHS is a potential therapeutic target and has been under preclinical tests [17]. Of the Indian hedgehog (IHH)/parathyroid hormone-related protein (PTHLH) pathway, induction of the PTHLH pathway and reactivation of bcl2 have been implicated in pathogenesis and progression of conventional CHS, and bcl2 was suggested to be a reliable marker for the distinction between low-grade CHS and enchondromas [18]. 

There is increase of genetic aberrations as CHSs progress from low to high grade. Although the role of *p53* in CHS pathobiology remains obscure, the presence of overexpression of the *p*53 protein, 17p1 alterations, and TP53 mutations mainly in almost all high-grade CHSs suggest that the *p*53 mutation is a late event involved in CHS progression [17, 19, 20]. Amplification of 12q13 and loss of 9p21 are some of the few consistent genetic aberrations found in conventional CHS. The 12q13 region harbors MDM2, a negative regulator of p53, and the 9p21 region harbors two cell cycle regulators, CDKN21/p16/INK4A and INK4A-p14^ARF^. The loss of INK4A/p16 expression was shown to be restricted to high-grade CHS, suggesting the role for CHS progression [17, 21].

### 2.6. Prognostic Factors

 For the prognosis of CHSs, the single most important predictor of local recurrence and/or metastasis is histological grade, although several histological and clinical parameters such as tumor necrosis, mitotic rate, type of surgery, and tumor location have been suggested to be associated with prognosis. Grade 1 tumor has indolent clinical behavior and no metastatic potential. The five-year survival by grade was 89% for patients with grade 1 and 57% for the combined group of patients with grades 2 and 3 tumors. Only high tumor grades (2 and 3) were significantly associated with the probability of metastasis [22, 23].

### 2.7. Treatment

CHS is considered relatively resistant to chemotherapy or radiotherapy, and the mainstay of treatment is surgical treatment. Wide, en bloc excision is the preferred surgical treatment in grade 2 or grade 3 CHS. In grade 1 CHS confined to the bone, extensive intralesional curettage followed by local adjuvant treatment and filling the cavity with bone graft has promising long-term clinical results and satisfactory local control [24].

### 2.8. CHSs in Specific Anatomic Locations

#### 2.8.1. CHSs of the Hands and Feet

**(1) Epidemiology**

The hands and feet are rare sites for central CHSs, whereas enchondromas are extraordinarily common in these sites. The median age of the patients at the time of diagnosis is 67 years (range, 21–87 years), with a slight preference for female in contrast with CHSs located in other sites of the skeleton [25, 26].

**(2) Sites of Involvement**

Occurrences in the hand are more common than in the foot, with the proximal phalanx affected most often. The fifth digit has the highest incidence of CHS, and the fourth digit is the least common site in the hands [27].

**(3) Clinical Features and Imaging**

Pain (usually without fracture) is usually present at presentation. The median size is approximately 3 cm (range, 1–8 cm). Radiologically, CHSs are predominantly lucent lesions, sometimes with areas of punctuate calcification, with irregular cortical destruction and extension into surrounding soft tissue.

**(4) Pathology**

The distinction between enchondroma and CHS is often difficult on histologic examination because enchondromas of the hands and feet can exhibit increased cellularity, binucleated cells, and hyperchromasia. Therefore, the most important histologic features of CHS in these sites are permeative growth pattern between preexisting bones and extension to soft tissue or joint space. Other histologic features suggesting malignancy are the presence of myxoid change and peripheral spindling of neoplastic chondrocytes.

**(5) Prognostic Factors**

CHSs of the hands and feet are typically low-grade CHSs with a propensity to recur, but a limited tendency to metastasize in contrast to CHSs located elsewhere and complete excision with margins of normal tissue is curative in almost all cases [25, 26]. CHSs of the calcaneus and the talus were more likely to metastasize.

#### 2.8.2. CHSs of the Craniofacial Region

**(1) Epidemiology and Sites of Involvement**

Craniofacial CHSs account for 2% of all CHSs and have a predilection for the skull base. The mean age of the patients at the time of diagnosis is 39 years (range, 10–79 years) (mean, 39 years). The temporo-occipital junction is the most preferred site in frequency followed by clivus and sphenoethmoid complex [28].

**(2) Clinical Features and Imaging**

Most patients present with symptoms related to the central nervous system. CT and MR imaging reveal bone destruction and associated soft tissue masses usually containing punctuate areas of chondroid mineralization [3].

**(3) Pathology**

The majority of the tumors are conventional CHSs of low to intermediate grade.

**(4) Prognostic Factors**

When the skull base CHS involves the clivus, distinction from chordoma is important because CHS has a much better prognosis than chordoma. Chordoma tends to occur in patients a decade older than do CHSs and grow much more rapidly. Skull base CHS has an excellent prognosis, and the 5- and 10-year disease-specific survival rates are reported to be both 99%. In contrast, the 5- and 10-year survival rates of chordoma have been reported to be approximately 51% and 35%, respectively [28].

## 3. Periosteal (Juxtacortical) CHS

Periosteal CHS is a rare malignant hyaline cartilage tumor arising from the external surface of bone and has also been referred to as parosteal CHS. 

### 3.1. Epidemiology and Sites of Involvement

 This tumor accounts for less than 2% of all CHSs and 0.2% of all bone tumors [29]. The tumor tends to affect younger adults than conventional CHS with peak incidences in the third to fourth decade of life. There is a slight male predilection. The most common site is the metaphyseal region of the long bones, especially the femur and the humerus.

### 3.2. Clinical Features and Imaging

 The clinical signs and symptoms are mostly nonspecific and present with pain or slowly growing mass. The lesion appears to involve the cortex with indistinct margins. On radiographs, the tumor often appears as a radiolucent juxtacortical soft tissue mass with sharply defined borders containing calcifications characteristic of cartilage tumors.

### 3.3. Pathology

 The lesion is usually large (mean size, 8.1 cm) and covered by a fibrous pseudocapsule that is continuous with the underlying pseudocapsule. The mass is usually round to oval, lobulated, and gritty white with areas of enchondral ossification and scattered calcification. Whereas periosteal osteosarcoma is commonly fusiform and shows less-constant chondroid features [30]. Histological features are similar to those of conventional CHS that is composed of solid nodules of hyaline cartilage with variable amount of myxoid stroma. Nodules of the tumor can invade surrounding soft tissues. Almost all periosteal CHS corresponds to grade 1 or 2 CHS. By definition, tumor osteoid should not be present within the tumor [30].

### 3.4. Differential Diagnosis

The differential diagnosis includes periosteal osteosarcoma. The peak age of incidence of periosteal osteosarcoma is 10 years younger than CHS. The most common anatomic site is the diaphysis or diaphyseal-metaphyseal area of the proximal tibia, followed by the femur and humerus. Periosteal osteosarcoma presents as small radiolucent lesions on the surface, with formation of spicules of bone perpendicular to the bone shaft. Histologically, periosteal osteosarcoma is intermediate grade, predominantly chondroblastic osteogenic sarcoma [31, 32]. Periosteal chondroma also should be distinguished from periosteal CHS. Periosteal chondroma is a slow-growing benign cartilaginous tumor arising within or under the periosteum. The size of the tumor is usually 1–3 cm in diameter. The peak incidence is in the second and third decades of life. The most common anatomic site is the metaphyseal region of long tubular bones. The proximal humerus is the most common site, followed by the femur and short tubular bones of the hands and feet. Histologically, the tumor consists of lobules of hyaline cartilage with foci of myxoid change. Although periosteal chondroma can be cellular and show binucleated chondrocytes, penetration into cancellous bone and nuclear anaplasia is not identified [33].

### 3.5. Prognostic Factors

 The prognosis for patients with periosteal CHS is favorable compared to that of intramedullary CHS. The overall 5-year metastasis-free survival is approximately 83%. The 5-year metastasis-free survival is less for patients with grade 2 tumors (50%) than for patients with grade 1 tumors (94%). Invasion of the medullary cavity is not frequent. Metastasis is exceptional and occurs very late [34]. Dedifferentiation has been rarely reported and is associated with poor prognosis [35].

## 4. Secondary CHS

Secondary CHS is a CHS arising in a benign precursor, either an osteochondroma or enchondroma. Although secondary CHS can be either central or peripheral, peripheral lesions are more common.

### 4.1. Peripheral Secondary CHSs

#### 4.1.1. Epidemiology

 Malignant transformation to peripheral CHS can be seen in 1% of solitary osteochondroma and 3~5% of patients with osteochondromatosis (hereditary multiple exostoses, HME) [36, 37]. HME is an autosomal dominant skeletal disease characterized by the formation of multiple cartilage-capped bone tumors growing outward from the metaphyses of long tubular bones.

#### 4.1.2. Genetics

**Figure 8:**
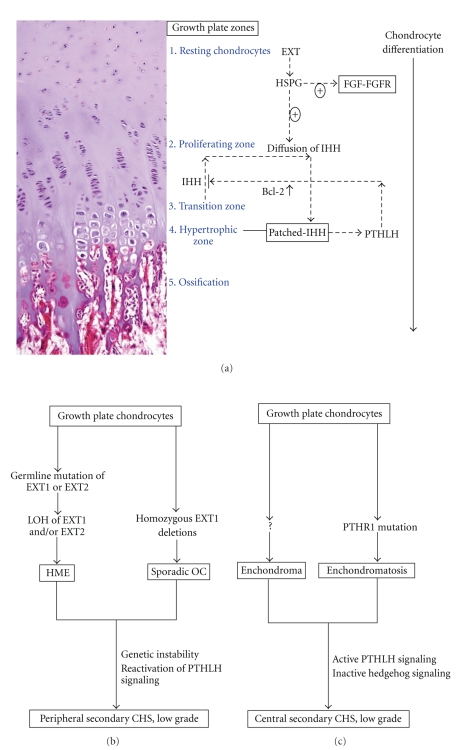
(a) Schematic representations of IHH/PTHLH signaling in the growth plate. The growth plate is composed of chondrocytes at different stages of differentiation, finally leading to longitudinal bone growth. This process is tightly regulated by IHH/PTHLH signaling. IHH, expressed by transition zone chondrocytes, diffuses and binds to Patched (Ptc) in the hypertrophic zone, stimulating PTHLH expression. PTHLH then binds to its receptor in the transition zone and upregulates Bcl-2, which inhibits chondrocyte differentiation and downregulates IHH secretion. EXT gene products play a role in the diffusion of hedgehog proteins and FGF-FGFR interaction. Therefore, defect or absence of EXT genes results in an abnormal IHH diffusion pattern, leading to an osteochondroma. (b) Proposed genetic model for peripheral secondary CHS. Analogous to Knudson's two-hit model, both alleles of an EXT gene are inactivated for osteochondroma formation in both HME and solitary osteochondroma. Genetic instability and reactivation of PTHLH signaling characterizes the malignant transformation of osteochondroma. (c) Proposed genetic model for central secondary CHS. Patients with enchondromatosis infrequently harbor PTHR1 mutation, which disrupts the normal IHH-PTHLH feedback loop, leading to constitutive hedgehog signaling. In most enchondromas, causative genetic or epigenetic changes have not been identified.

 HME is caused by mutations in either of two genes: exostosin-1 (EXT1), which is located on chromosome 8q24.11–q24.13, and exostosin-2 (EXT2), which is located on chromosome 11p11-12. Most of the mutations in these two genes are inactivating mutations (nonsense, frame shift, or splice-site mutations), causing premature termination of the EXT proteins and the loss of protein function [38, 39]. In accordance with Knudson's two-hit model, both alleles of EXT seem to need to be inactivated for osteochondroma formation. Loss of heterozygosity (LOH) of EXT1 and/or EXT2 is shown in some solitary osteochondromas and HMEs (Figures 8(a) and 8(b)) [40, 41]. The absence of LOH in a proportion of osteochondromas seems to be because cartilaginous cap of osteochondroma is mosaic. Therefore, detection of a second mutational event depends on the balance between EXT mutated and wild-type cells [42, 43].

In the growth plate, IHH regulates chondrocyte proliferation and differentiation in a tightly regulated paracrine feedback loop, together with PTHLH, and deregulated IHH signaling has been implicated in the pathogenesis of osteochondromas. The EXT genes encode glycosyltransferases involved in the biosynthesis of heparan sulfate (HS) chains at HS proteoglycans (HSPGs). HSPGs have been shown to play a role in the diffusion of IHH, PTHLH, and fibroblast growth factor (FGF), all of which are involved in chondrocyte proliferation and differentiation. Therefore, EXT inactivation affects hedgehog signaling by defective HS (Figures 8(a) and 8(b)). In addition, disturbed hedgehog signaling can cause defect in the body collar because hedgehog is important for the formation of the bony collar. EXT^−/−^ cells lose their ability to respond to polarity signals, then grow out of the bone, and then recruit normal cells to form an osteochondroma. IHH/PTHLH and FGF signaling molecules are mostly absent in osteochondromas and reexpressed with the progression of osteochondroma towards peripheral CHSs. Upregulation of PTHLH and Bcl-2 characterizes malignant transformation of osteochondroma [42, 44–46].

#### 4.1.3. Clinical Features and Imaging

**Figure 9:**
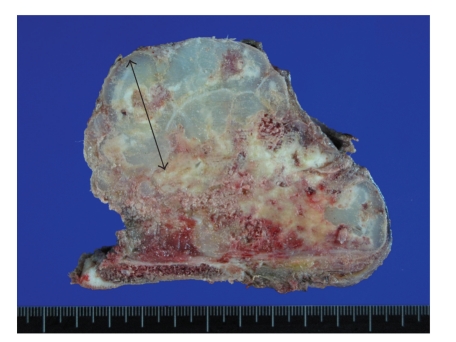
Secondary CHS arising in osteochondroma. Note the thickening of cartilage cap (2.5 cm, arrows).

 In osteochondromas, lesions that continue to grow or cause pain after skeletal maturity suggest malignant transformation since osteochondromas only rarely grow after skeletal maturation. A thick hyaline cartilage cap greater than 1.5~2.0 cm thick (in osteochondroma: 6 to 8 mm thick) in a skeletally mature patient has been cited as a sign of possible malignant transformation (Figure 9). However, the key for the diagnosis is the histopathologic differentiation of the cartilaginous proliferation. Radiographic findings that suggest malignancy are growth of a previously unchanged osteochondroma in a skeletally mature patient, irregular or indistinct lesional surface, focal regions of radiolucency in the interior of the lesion, erosion or destruction of the adjacent bone, and a soft tissue mass with scattered or irregular calcifications. Malignant transformation develops earlier in patients with HME (average, 25~30 years) than in those with solitary osteochondroma (average, 50~55 years). Malignant transformation before the age of 20 is very unusual [37, 47].

#### 4.1.4. Pathology

**Figure 10:**
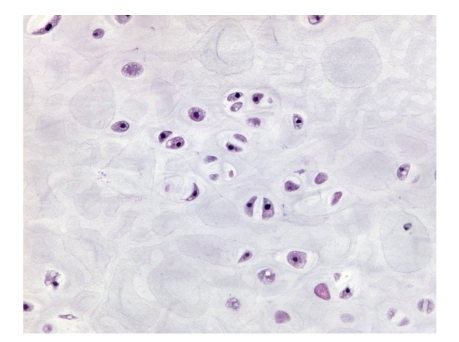
Grade 1 CHS arising in osteochondroma with loss of organized architecture and mild nuclear atypia.

CHS arising in osteochondroma is usually solitary and low grade in type, but multifocality and dedifferentiation have also been reported (Figure 10). On microscopic examination, loss of cartilaginous columnar architecture, fibrous bands between cartilage lobules, increased nuclear atypia, mitosis, or myxoid changes are features suggestive of malignant transformation.

#### 4.1.5. Prognostic Factors and Treatment

 Malignant transformation of osteochondroma is usually treated with surgery. Treatment of patients with HME is more complex than that of patients with solitary osteochondroma. Because most of these lesions are low-grade CHS, the overall prognosis is good, with long-term survival in 70~90% of patients. Local recurrence rate varies with adequacy of the tumor margins, from 0–15% in widely resected cases to 57~78% in cases with marginal or intralesional resection [37, 48]

### 4.2. Central Secondary CHSs

#### 4.2.1. Epidemiology

 Central secondary CHSs develop as malignant transformation of enchondroma (extremely rare) or enchondromatosis such as Ollier disease or Maffucci syndrome. Patients with Ollier disease and Maffucci syndrome have a 25~30% risk of developing CHS.

#### 4.2.2. Genetics

 The exact cause of Ollier disease and Maffucci syndrome remains to be elucidated, although mutation in the PTHR1 gene, c.448C>T (p.R150C), has been suggested to cause enchondromatosis [49, 50]. A mutation in PTHR1 disrupts the normal IHH-PTHLH feedback loop, causing constitutive hedgehog signaling (Figure 8(c)).

#### 4.2.3. Clinical Features and Imaging

 Ollier disease is a nonhereditary developmental abnormality characterized by multiple enchondromas throughout the epiphyses, metaphyses, and diaphyses of the skeleton. The size, number, location, and evolution of enchondromas are quite variable. Clinically, Ollier disease often shows asymmetric, unilateral involvement of the lower extremities, but it is often bilateral in the hands and feet. Any portion of the skeleton formed by endochondral ossification can be affected; however, Ollier disease rarely affects bones formed by membranous ossification, such as the skull and facial bones. Maffucci syndrome is a condition in which enchondromatosis is associated with soft tissue hemangiomas [51, 52].

The development of pain as well as the appearance of soft tissue mass, areas of bone destruction, endosteal scalloping, periosteal reaction, and fracture without significant trauma raises the suspicion of malignant transformation of enchondromatosis.

#### 4.2.4. Pathology

 CHS arising in enchondromatosis is usually a low-grade tumor like that of osteochondroma or HME. Therefore, identification of invasion to surrounding tissues or marked myxoid change is helpful to the diagnosis.

#### 4.2.5. Prognostic Factors and Treatment

 CHS in enchondromatosis has the same prognosis as conventional CHS and depends on the site and grade of the tumor. Malignant transformation of enchondromatosis is greater in Maffucci syndrome than Ollier disease, and the prognosis is worse than that of Ollier disease.

## 5. Dedifferentiated CHS

**Figure 11:**
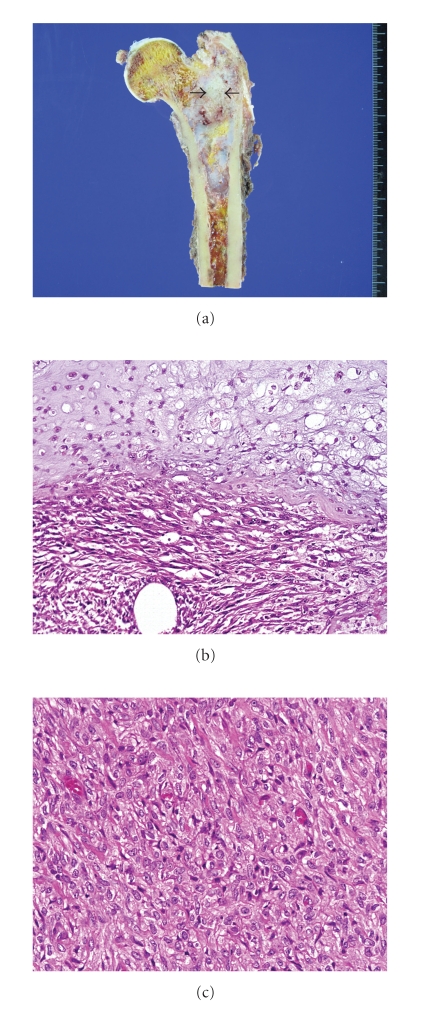
(a) An ill-demarcated lobulating firm mass (8.5 × 4.0 × 4.0 cm) in the proximal femur. Most of the mass which is white to bluish gray is conventional chondrosarcoma. The yellowish gray area in the center (arrows) is dedifferentiated area. (b) The abrupt transition between conventional CHS and dedifferentiated component. (c) The dedifferentiated component consisting of malignant spindle cells without matrix formation (malignant fibrous histiocytoma).

Dedifferentiated CHS is a distinct variant of CHS containing a well-differentiated cartilage tumor, either an enchondroma or a low-grade CHS, with an abrupt transition to foci having high-grade noncartilaginous sarcoma (Figures 11(a)–11(c)). 

### 5.1. Epidemiology and Sites of Involvement

 Dedifferentiated CHS makes up 10% of CHSs. The average age of presentation is between 50 and 60 years. Men and women are affected equally. The most common affected sites are the femur and pelvis [53, 54]. The majority of lesions occur centrally in the medullary cavity of bone, although there are reports of dedifferentiation in juxtacortical CHS or from preexisting osteochondroma [35, 55].

### 5.2. Clinical Features and Imaging

 The patients present most frequently with pain (90%), followed by pathologic fracture and soft tissue mass. The proportion of noncartilaginous component varies greatly and may be frequently osteosarcoma, fibrosarcoma, or malignant fibrous histiocytoma. Rhabdomyosarcoma, leiomyosarcoma, and angiosarcoma have been reported as the dedifferentiated component. Dedifferentiated CHSs have a wide range of radiological appearance; however, the presence of “tumoral dimorphism” with cartilaginous component and aggressive lytic component invading adjacent soft tissues suggests a diagnosis of dedifferentiated CHS [56].

### 5.3. Histogenesis

 There are at least three hypotheses explaining the origin of dedifferentiated CHS. One theory is that the high-grade noncartilaginous tumor component arises in a long-standing low-grade cartilaginous tumor, particularly when the tumor is recurrent. The second hypothesis is that noncartilaginous component arises simultaneously with CHS with ability to differentiate. The third theory is that noncartilaginous sarcoma represents malignant transformation of adjacent inflamed but otherwise normal tissue [53].

### 5.4. Genetics

 So far, no specific aberrations seem to be associated with dedifferentiated CHS, although dedifferentiated component tends to show aneuploidy, loss of heterozygosity, and amplification and deletion more frequently [57]. Recently an array-based comparative genomic hybridization (Array-CGH) study demonstrated statistically significant association between high-grade tumor (grade III and dedifferentiated) and the recurrent genetic deletions at 5q14.2~q21.3, 6q16~q25.3, 9p24.2~q12, and 9p21.3 [58]. Regarding dedifferentiated peripheral CHSs, dedifferentiated component shows more frequent expression of cyclin D1, p53, plasminogen activator inhibitor 1 (PAI-1), and CD44. The PTHLH signaling seems to be downregulated in chondrogenic component of dedifferentiated peripheral CHSs whereas FGF signaling pathway in active, compared with secondary peripheral CHSs without dedifferentiation [59].

### 5.5. Prognostic Factors

 Because the dedifferentiated component determines the prognosis, its identification is a key for management. In spite of aggressive treatment, the overall survival rate is less than 10% at five years, with a median survival time of 7.5 months. While local control is achieved in the majority of cases, distant disease remains the greatest clinical challenge, developing in 90% of patients [60].

## 6. Mesenchymal CHS

Mesenchymal CHS is a rare highly malignant tumor that arises in bone but can occur in extraskeletal sites and is characterized by highly cellular areas composed of undifferentiated small round or spindle cells admixed with lobules of mature hyaline cartilage. 

### 6.1. Epidemiology

Mesenchymal CHS makes up less than 2~13% of all primary CHSs. Mesenchymal CHS occurs at any age, with peak incidences in the second to third decades of life. There is no significant sex predilection [61].

### 6.2. Sites of Involvement

 The skeletal tumors show a widespread distribution. The craniofacial region is the most frequently affected site (15~30%), specifically the mandible and maxilla. Other common sites include femur, ribs, spine, pelvis, and humerus [1, 3]. About 7% of the osseous lesions are reported to be multicentric. Up to one-third of the lesions primarily affect extraskeletal sites. The meninges (cranial > spinal) are the most common sites of extraskeletal involvement, followed by lower extremity [61].

### 6.3. Clinical Features and Imaging

 Most patients presented with pain and/or swelling. Duration of symptoms prior to the histologic diagnosis is quite variable, ranging from few days to several years. Oncogenic osteomalacia secondary to mesenchymal CHS has been reported.

Roentgenographically, mesenchymal CHSs in bone frequently resemble ordinary CHSs, showing osteolytic and destructive appearances with stippled calcifications. Tumors in extraskeletal sites are almost always identified as a mass with flocculent or stippled calcific densities. Sclerosis or periosteal reaction is uncommon, while expansion of the bone, cortical destruction, or cortical breakthrough with extraosseous extension of soft tissue is common [61].

### 6.4. Pathology

#### 6.4.1. Gross Findings

 Grossly, the tumors are gray to tan, firm to soft, and usually well defined and well circumscribed. The size of the tumor ranges from 3 cm to 30 cm in diameter. Lobulation is infrequent. Most lesions contain hard mineralized deposits that vary in amount from scattered foci to prominent areas. Some tumors show a clearly cartilaginous appearance, even in a small area. Necrosis is uncommon but may be prominent [62]. Bony expansion with cortical thinning or bone destruction and soft tissue invasion is frequent.

#### 6.4.2. Microscopic Findings

**Figure 12:**
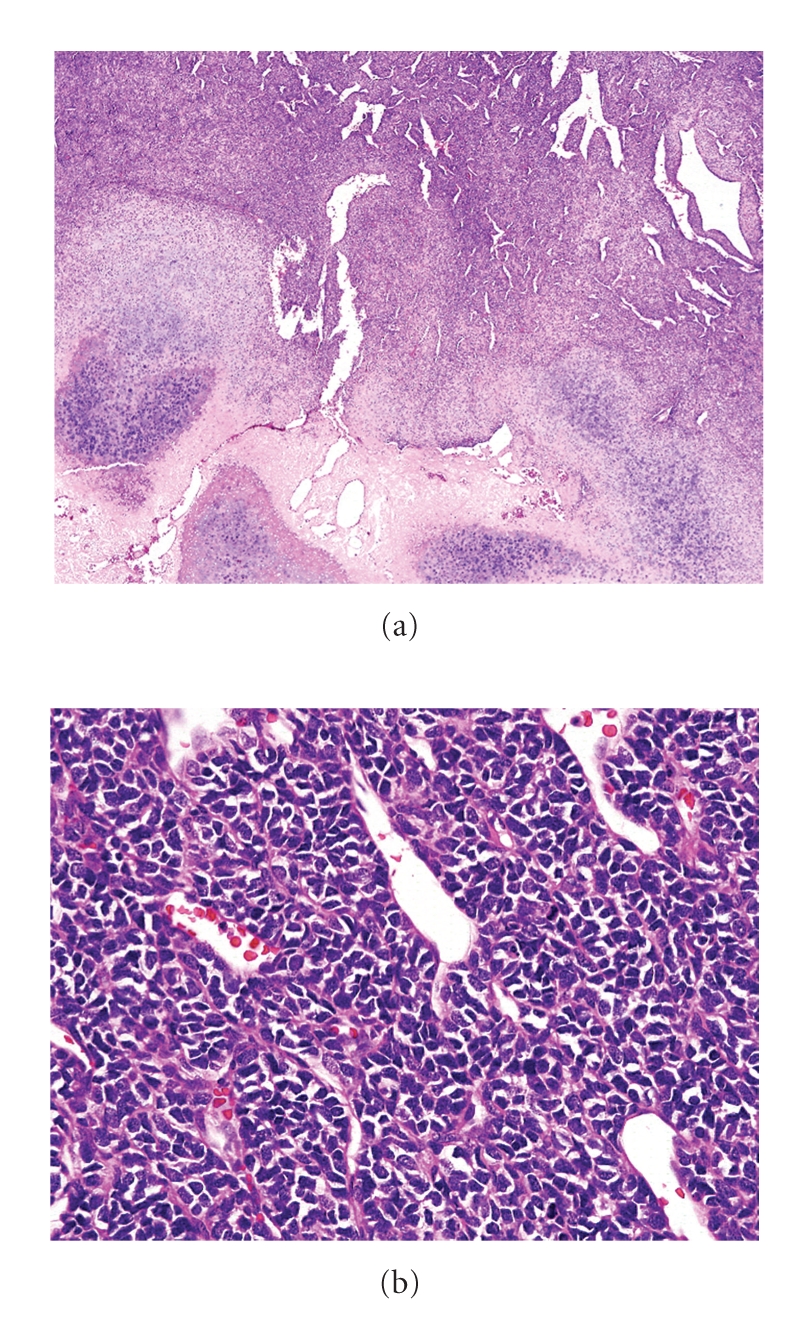
(a) Mesenchymal CHS showing bimorphic pattern, consisting of islands of hyaline cartilage and undifferentiated cells. Note hemangiopericytomatous vascular pattern. (b) The undifferentiated small cells with relatively uniform oval to round nuclei and scanty amount of cytoplasm.

 Histologically, a bimorphic pattern with cellular zones of undifferentiated small or spindle cells and islands of hyaline cartilage is pathognomonic (Figure 12). The amount of cartilage is highly variable. Transition from cellular areas to zones with hyaline cartilage is usually abrupt but can be gradual. The undifferentiated cells with oval nuclei frequently tend to be arranged in a vague alveolar pattern or in solid sheets, resembling Ewing sarcoma. A hemangiopericytomatous vascular pattern is seen in most cases. Osteoclastic giant cells can be seen, usually adjacent to the cartilaginous islands [61].

#### 6.4.3. Immunohistochemical Findings

**Figure 13:**
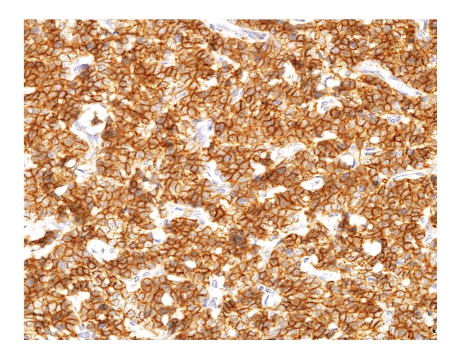
The undifferentiated small cell component in mesenchymal CHS strongly positive for CD99.

 Immunohistochemically, the cartilaginous area is strongly positive for S-100 protein, whereas only scattered single cells in the undifferentiated areas stain for this antigen. The undifferentiated small cell component of mesenchymal CHSs is consistently positive for CD99 and may stain for vimentin and Leu7 while negative for osteocalcin, actin, cytokeratin, and epithelial membrane antigen (EMA) (Figure 13). SOX9 is almost invariably positive in both components [63, 64].

### 6.5. Genetics

 At present, cytogenetic findings of mesenchymal CHS are rarely reported. An identical Robertsonian translocation involving chromosomes 13 and 21 (der(13;21)(q10;q10)) has been detected in two cases of mesenchymal CHSs, possibly representing a characteristic rearrangement for this histopathologic entity [65]. The t(11;22) of the Ewing family of tumors is not seen in mesenchymal CHS. Although approximately 60% of the tumors demonstrate p53 overexpression, no mutation has been found within exons 5~9 regions [66].

### 6.6. Prognostic Factors

 The prognosis of mesenchymal CHS is poor. However, the clinical course may be protracted. Because local recurrence or metastasis sometimes is encountered even after more than 20 years, long-term follow-up is essential. The 5-year survival rate was 54.6%, and the 10-year survival rate was 27.3% in a group of 23 patients from the Mayo Clinic. The most frequent site of metastasis is the lung. Ablative surgical treatment seems to be the treatment of choice [61].

## 7. Clear Cell CHS

Clear cell CHS is a rare, low-grade malignant tumor characterized by clear cytoplasm of the tumor cells. 

### 7.1. Epidemiology and Sites of Involvement

 The tumor accounts for about 1-2% of all CHSs. The lesion affects males more commonly than females (2.6 : 1) and has a predilection for the end of long bones (epiphysis) in contrast to conventional CHS which tends to occur in the meta-diaphyseal regions of the bone. Although the proximal femur and humeral head are the sites most commonly affected in about two-thirds of the cases by this lesion, most bones including spine, rib, pelvis, and hands and feet can be involved. The age range is wide with peak incidences in the third to fourth decades of life [67].

### 7.2. Clinical Features and Imaging

 Clinically, clear cell CHS presents one or two decades later than chondroblastoma. The clinical symptoms are nonspecific; however, pain is the most common presenting symptom. More than half of patients have pain for longer than a year. 

**Figure 14:**
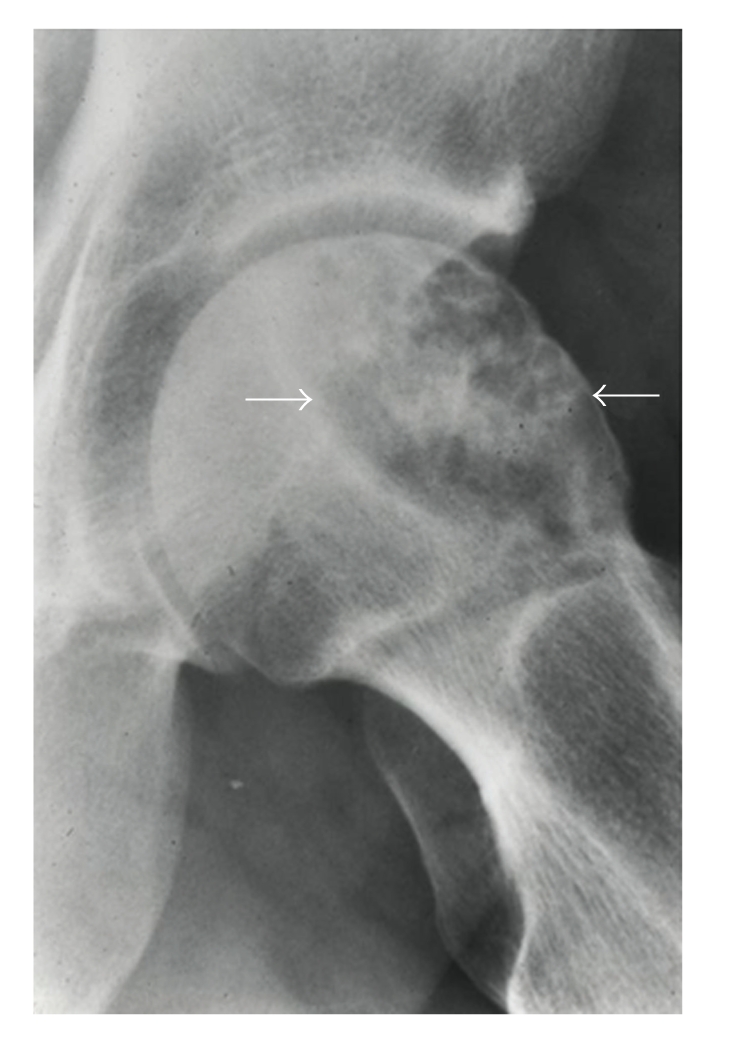
Clear cell CHS located in the epiphysis metaphysis of the proximal femur. The lesion is lytic, slightly expansile, and well delineated from the adjacent normal bone.

Roentgenographically, the lesion is typically located in the epiphysis metaphysis of long bone. The lesion is most often purely lytic and slightly expansile, with a sharp margin between the tumor and the adjacent normal bone (Figure 14). Typically, there is no cortical destruction or periosteal new bone formation. More than one-third of the long bone lesions contained matrix mineralization with a characteristic chondroid appearance. Pathologic fracture is occasionally present. Flat bone lesions are typically lytic and expansile with occasionally demonstrated areas of cortical disruption. Typically, matrix mineralization, when present, is amorphous. Adjacent bone marrow edema is typically absent or only minimally observed [68].

### 7.3. Pathology

**Figure 15:**
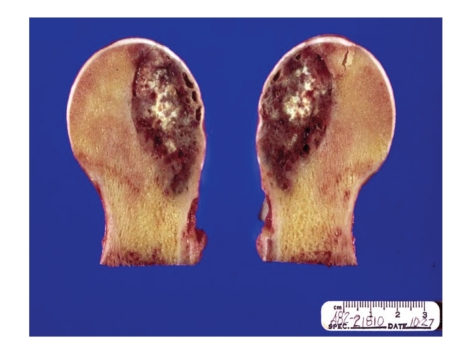
Gross finding of clear cell chondrosarcoma in the proximal femur.

**Figure 16:**
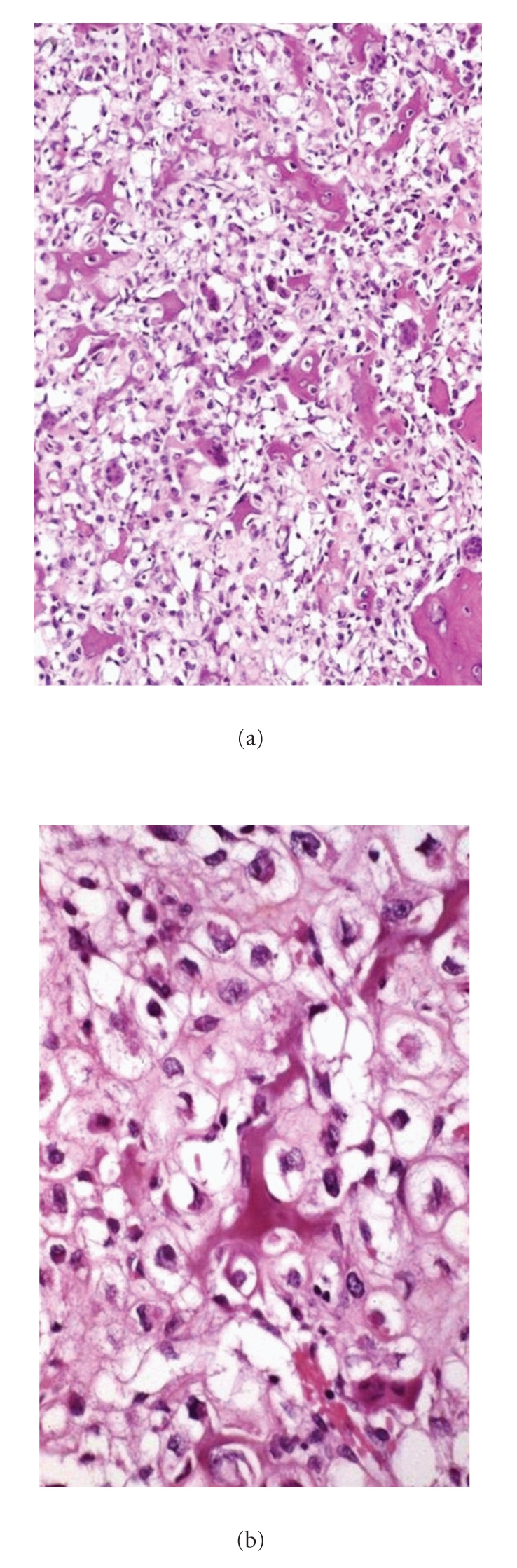
(a) Tumor cells with clear cytoplasm between irregular trabeculae of woven bone. (b) Higher magnification of tumor cells with abundant clear or faintly granular cytoplasm and central round nuclei containing occasional prominent nucleoli.

 Grossly, the tumors are well circumscribed and may be either firm or soft. Grossly cartilage is not usually present (Figure 15). The lesions consist of clear cells arranged in an indistinct lobular pattern and having round, large, centrally located nuclei with clear cytoplasm and distinct cytoplasmic membranes (Figure 16). Clear cell components in clear cell CHS are accompanied by “conventional” foci of CHS in less than 50% of cases. Secondary findings including areas of osteogenesis, osteoclast-like giant cells, and zones resembling aneurysmal bone cyst or giant cell tumor of bone could be found. Mitotic figures are rare [69]. Dedifferentiation to high-grade sarcoma has been rarely reported [70]. Clear cell CHS should be differentiated from other osseous tumors which can show focal or diffuse clear cell changes such as osteosarcoma, chondroblastoma, chordoma, adamantinoma, and Ewing's sarcoma/primitive neuroectodermal tumor as well as metastatic renal clear cell carcinoma. The clear cells are positive for type II collagen as well as S-100 protein and aggrecan [71].

### 7.4. Genetics

 Recent molecular genetic studies show that genetic alterations of p53 are infrequent in clear cell CHS in spite of substantial overexpression of p53 [72]. Tumor-specific cytogenetic change is currently unknown; although a case report described clonal chromosomal abnormalities in three of four cases of clear cell CHS [73].

### 7.5. Prognostic Factors

 Clear cell CHS is a low-grade malignancy and usually curable by en bloc resection. About 25% of patients experience local recurrences or metastases. However, tumor-related death is uncommon, particularly when the lesion has been completely resected en bloc [69].

## Figures and Tables

**Table 1:** Classification of CHSs.

Anatomic classification	
Intramedullary (central)	
Peripheral	
Juxtacortical (periosteal)	
Primary versus secondary	
Primary	
Arise de novo	
Secondary	
Arise in a benign precursor, either an osteochondroma or enchondroma Arise in a benign precursor, either an osteochondromatosis or enchondromatosis	
Histologic classification	
Conventional	
Dedifferentiated	
Mesenchymal	
Clear cell	

**Table 2:** Summary of frequency, age, sex, and prognosis of CHSs.

Subtype	Frequency	Gender (M : F)	Peak age	5-year survival
Conventional	85%	1.5–2 : 1	40–60	G1:89%, G2, and G3:57%
Periosteal	<2%	Slight male predilection	20–30	83%
Dedifferentiated	10%	1 : 1	50–60	<10%
Mesenchymal	<2–13%	1 : 1	10–20	54.6%
Clear cell	1-2%	2.6 : 1	20–30	>80%
